# Postoperative Pain Trajectories After Craniotomy in Brain Tumor Patients: A Cross-Sectional Study of Intensity and Characteristics

**DOI:** 10.3390/medicina61091661

**Published:** 2025-09-13

**Authors:** Biljana Filipović, Paško Berišić, Snježana Čukljek, Irena Kovačević, Martina Smrekar, Ana Marija Švigir

**Affiliations:** 1 Department of Nursing, University of Applied Health Sciences, Mlinarska 38, 10000 Zagreb, Croatia; snjezana.cukljek@zvu.hr (S.Č.); irena.kovacevic@zvu.hr (I.K.); martina.smrekar@zvu.hr (M.S.); anamarija.svigir@zvu.hr (A.M.Š.); 2 Department of Nursing, Faculty of Health Studies, University of Rijeka, Viktora Cara Emina 5, 51000 Rijeka, Croatia; 3 Neurosurgery Department, University Hospital Centre Zagreb, Mije Kispatica 12, 10000 Zagreb, Croatia; pasko.berisic2000@gmail.com

**Keywords:** postoperative pain, craniotomy, brain tumors, pain management

## Abstract

*Background and Objectives:* Post-craniotomy pain is common in brain tumor patients, yet the day-to-day course and qualitative features during the first postoperative days are insufficiently described; prior reports often mix heterogeneous surgical indications or focus only on the immediate PACU period or long-term headache. We aimed to address this gap by quantifying early postoperative pain trajectories and characterizing pain quality and timing in a homogeneous brain-tumor cohort. *Materials and Methods:* In this cross-sectional study of 100 adults after craniotomy, pain intensity (0–10 NRS) and pain characteristics were assessed on postoperative days (POD) 2–4 using a structured questionnaire; subgroup analyses compared first-time vs. previously operated patients. *Results:* Median NRS decreased from 2.00 on POD2 to 0.00 on POD4 (Friedman *p* < 0.001). Pulsatile pain was most frequent (≈46% on POD2), while higher intensities were uncommon by POD4; no significant differences were observed between first-time and previously operated patients. *Conclusions:* These findings fill a short-term evidence gap by delineating early pain trajectories and descriptors specific to brain-tumor craniotomy and support pragmatic, individualized analgesia in the first postoperative days. Future studies should complement NRS with multidimensional outcomes to capture affective and functional aspects of pain.

## 1. Introduction

Craniotomy is a highly advanced neurosurgical procedure that involves the removal of a portion of the skull to provide direct access to the brain. It is widely used for the diagnosis and treatment of various brain pathologies, including tumors, vascular diseases, epilepsy, and the consequences of traumatic brain injuries [1,2]. While the primary objectives of craniotomy include reducing intracranial pressure, excising abnormal tissue, and enhancing neurological function, the procedure is frequently accompanied by significant postoperative pain, which can adversely affect patient recovery [3,4]. It is crucial to acknowledge the specific anatomical structures that act as primary pain generators during the procedure. The skin and dura mater are sensitive to nociceptive stimuli. Surgical incisions through the skin and subsequent incision or manipulation of the dura mater are typically associated with the most intense nociceptive stimulation. Such intraoperative events play a significant role in the patient’s postoperative pain experience [5].

Postoperative pain following craniotomy is a multifactorial phenomenon involving complex physiological and psychological mechanisms. Patients commonly describe the pain as pulsatile or pressure-like headaches, often resembling tension-type headaches, which can emerge within hours of the procedure and persist for days [6]. Incisional pain and neuropathic pain, resulting from nerve damage or dysfunction during surgery, are also frequently reported [7]. Alarmingly, studies indicate that up to 22.5% of patients experience persistent pain even one year after craniotomy, highlighting the chronic impact of this condition [6].

The severity and characteristics of post-craniotomy pain are influenced by various factors, including surgical trauma, tissue manipulation, skull drilling, and stretching of the dura mater. These processes trigger inflammatory responses, nociceptor activation, and central sensitization [8,9]. Additionally, psychological factors such as preoperative anxiety can further amplify pain perception [10]. In patients with brain tumors, the type and location of the tumor, as well as the extent of surgical intervention, are particularly significant in shaping the postoperative pain experience.

Brain tumors are a heterogeneous group of pathologies, classified by histological and molecular characteristics, enabling precise diagnosis and targeted therapeutic approaches [11]. These classifications also inform the complexity of surgical interventions, as tumor type and location often pose unique challenges for neurosurgeons. Despite significant progress in understanding tumor biology, limited research has explored the relationship between tumor characteristics and postoperative pain, making this an area of clinical and scientific importance. Recent studies have highlighted that while the type, location, and size of brain tumors may not consistently predict headache occurrence, certain tumor locations are associated with characteristic headache syndromes [5]. For example, metastatic tumors to the skull base and other areas can cause distinct clinical syndromes such as orbital headaches with diplopia and ptosis, parasellar headaches with ocular paresis, and severe occipital pain from occipital condyle tumors. Furthermore, conditions such as trigeminal autonomic cephalgias and pituitary apoplexy show specific headache profiles, underscoring the complex relationship between tumor characteristics and headache manifestations [12].

Effective management of postoperative pain following craniotomy requires a combination of pharmacological and non-pharmacological approaches. Traditional pharmacological interventions include opioids, nonsteroidal anti-inflammatory drugs, local anesthetics, and gabapentinoids, each with specific benefits and limitations [13]. Concurrently, non-pharmacological methods such as physical therapy and psychological support can further alleviate pain and reduce reliance on analgesics [14,15]. Although post-craniotomy pain has been described, critical gaps remain for brain-tumor populations: (i) day-by-day characterization beyond the immediate postoperative period is limited; (ii) qualitative descriptors and timing of pain onset in the early days are under-reported; and (iii) prior craniotomy status is seldom examined within a homogeneous tumor cohort. To address these gaps, we evaluated pain intensity and features on POD2–POD4 after craniotomy for brain tumors and explored differences by surgical history, providing granular short-term data to inform early postoperative pain management.

## 2. Materials and Methods

### 2.1. Design

This study was conducted as a cross-sectional, non-interventional study in Zagreb, Croatia.

### 2.2. Participants

**Figure 1:**
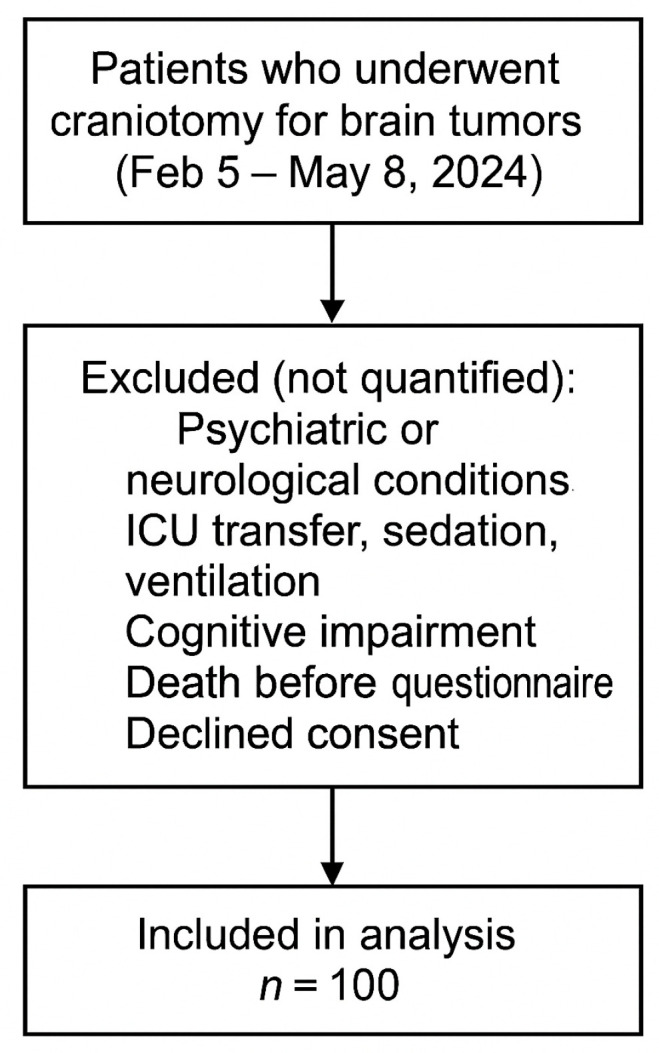
Flowchart of patient inclusion.

All adult patients who underwent craniotomy for brain tumors at the Clinical Hospital Center Zagreb between 5 February and 8 May 2024 were consecutively approached for inclusion in the study. Patients were included if they met the eligibility criteria and provided informed consent. Patients who did not meet the inclusion criteria (e.g., psychiatric or neurological conditions affecting pain perception, ICU transfer, sedation, cognitive impairment, or death before questionnaire completion) were excluded from the study. While all eligible patients were approached during the study period, specific numbers of excluded individuals were not tracked, as the study design prioritized reaching the predefined sample size of 100 participants (Figure 1).

Patients were additionally categorized into two subgroups according to their surgical history: those with a previous craniotomy and those undergoing their first craniotomy. This categorization was applied to examine whether prior surgical experience was associated with differences in postoperative pain intensity or characteristics.

Preoperative analgesia was not routinely administered. Patients experiencing discomfort were consulted about their personal pain management preferences, specifically regarding analgesics they commonly use at home. This approach allowed for individualized pain management, aligning with patient familiarity and preference, albeit not in a structured preoperative regimen.

Following surgery, patients were administered a unimodal postoperative analgesic regimen, including metamizole sodium at a dose of 2.5 g intravenously, ketoprofen at 100 mg administered either intravenously or orally, or paracetamol at 1000 mg also administered via either route, all on an intermittent basis. This regimen was selected based on its proven effectiveness and tolerability within this patient cohort, aimed at optimizing pain control while minimizing the potential for adverse effects.

### 2.3. Data Collection

Data collection was performed daily from the second to the fourth postoperative day. Postoperative Day 1 was defined as the first day after surgery. Pain assessment commenced on Postoperative Day 2 to avoid the potential influence of residual anesthesia on pain perception and self-reporting. Patients independently assessed and reported pain intensity and therapeutic response using a structured questionnaire. The numerical rating scale (NRS; 0 = no pain, 10 = worst possible pain) was applied within the questionnaire to evaluate pain intensity. Nurses facilitated the data collection process, assisted patients when necessary, and ensured that all responses were complete and accurately recorded.

This questionnaire was administered exclusively to conscious, communicative patients capable of self-reporting pain. Patients who were transferred to the ICU postoperatively, sedated, ventilated, cognitively impaired, or who died before completing the questionnaire were excluded from the study.

### 2.4. Instrument

The data collection questionnaire was specifically developed by researchers at the University of Applied Health Sciences, Zagreb, Croatia, to enable structured and standardized acquisition of key variables relevant to the research objectives. The questionnaire included demographic information (e.g., gender, age, marital status, occupation, educational level, and smoking status), clinical characteristics (type and location of the brain tumor), postoperative pain features (pain intensity, pain characteristics, and factors influencing pain fluctuations), and details regarding pain management practices (type of analgesics used, route of administration, timing, and dosage).

The numerical rating scale (NRS; 0 = no pain, 10 = worst possible pain) was incorporated within the questionnaire to ensure a standardized assessment of pain intensity.

### 2.5. Statistics

Statistical analyses were performed using IBM SPSS Statistics for Windows, Version 26.0 (IBM Corp., Armonk, NY, USA), applying both descriptive and inferential statistical methods. Descriptive measures included mean, standard deviation, median, and interquartile range, summarizing demographic and clinical characteristics of the patient sample. Inferential statistics involved *t*-tests to compare post-surgery pain intensities between operated and previously non-operated groups, and χ^2^ tests to investigate associations between categorical variables like tumor type and location.

The Friedman test was applied to evaluate temporal changes in pain intensity over measured time, suitable for the study’s non-parametric data. Statistical significance was set at *p* < 0.05, ensuring that findings were robust and reliable for clinical interpretation.

### 2.6. Ethics

This research was approved by the Ethics Committee of the Clinical Hospital Center Zagreb, Croatia on 8 January 2024 (Class: 8.1-24/6-2; No.: 02/013 AG). Before participation, the objectives, procedures, potential risks, and benefits of the study were clearly explained to the patients. Informed consent was obtained in written form, ensuring that all participants were aware that they could withdraw from the study at any time without affecting their subsequent medical care.

## 3. Results

Sociodemographic characteristics of the study participants are presented in Table 1. The sample consisted of an almost equal distribution of males and females, predominantly aged between 30 and 60 years, with the majority being married, employed, and having completed secondary education.

Table 2 illustrates the relationship between the type and location of brain tumors and the history of prior operations. Gliomas are the most common type of tumor, constituting 40% of all cases, with an equal proportion among operated (36.1%) and previously non-operated patients (50%). Meningiomas follow, present in 32% of participants, with a higher proportion among operated patients (36.1%) compared to previously non-operated ones (21.4%). Metastatic brain tumors are the third most common (18%), while other types of tumors, such as vestibular Schwannoma (6%) and individual cases of pituitary adenoma, medulloblastoma, craniopharyngioma, and hemangioblastoma make up less than 2% of the sample. Primary brain lymphomas and tumors of the pineal region were not recorded. The most frequent tumor locations are the frontal lobe (28%) and temporal lobe (24%), while tumors in the parietal lobe (16%), cerebellum (8%), and occipital lobe (7%) have lesser representation. Combined tumor locations, including the fronto-parietal (4%) and parieto-occipital regions (5%), were also observed. Statistical analysis indicates that there are no significant differences in the frequency of tumor types (*p* = 0.112) nor in their locations (*p* = 0.462) between operated and previously non-operated patients.

On the second day post-craniotomy, 40.3% of operated and 42.9% of previously non-operated patients reported no pain. The majority of patients who experienced pain rated its intensity as low to moderate (1–5), with no significant difference between groups (*p* = 0.894). Pulsatile pain was the most frequently reported characteristic (46.5% of operated and 43.8% of previously non-operated), while other types of pain, such as dull (14% versus 12.5%) and sharp (11.6% versus 12.5%), were less prevalent. No statistically significant difference was found in pain descriptions (*p* = 0.053), although shooting pain was more commonly reported by previously non-operated patients (25% versus 2.3%). On the second day post-craniotomy, unexpected pain was reported by 55.8% of operated and 31.3% of previously non-operated patients. This higher incidence in operated patients could be attributed to the immediate postoperative response to surgical trauma, where pain can often arise unpredictably as the effect of anesthesia diminishes. Unexpected pain in previously non-operated patients may reflect individual variations in pain threshold and response to the stress of surgery. Pain at rest was noted in 25.6% of operated and 31.3% of previously non-operated, while pain during movement was reported in 14% of operated and 37.5% of previously non-operated patients. No significant difference was observed in the timing of pain onset between groups (*p* = 0.240) (Table 3).

On the third day post-craniotomy, 37.5% of operated and 46.4% of previously non-operated patients reported no pain. The most common pain intensity was level 1, reported by 16.7% of operated and 17.9% of previously non-operated patients, while intensities 2 and 3 were reported with similar frequency in both groups. Higher intensities of pain (4 and above) were rare, and differences between groups were not statistically significant (*p* = 0.929). Pulsatile pain remained the most commonly reported pain characteristic in both groups (44.4% of operated and 40% of previously non-operated), while dull and wandering pains were reported by 13.3% of patients in both groups. Shooting pain was more frequent among previously non-operated (33.3% versus 8.9% operated), while burning and sharp pains were almost exclusively noted among operated patients. Differences in pain descriptions between groups were not statistically significant (*p* = 0.350). Pain most frequently occurred unexpectedly (66.7% operated and 33.3% previously non-operated). Pain at rest was recorded in 4.4% of operated and 20% of previously non-operated, while pain during movement was reported in 22.2% of operated and 46.7% of previously non-operated patients. No significant differences were noted in the timing of pain onset between the two groups (*p* = 0.067) (Table 4).

On the fourth day post-craniotomy, 50% of operated and 53.6% of previously non-operated patients reported no pain. The most common pain intensity was level 1, with rates of 19.4% in the operated group and 21.4% in the previously non-operated. Pain intensities of 2 and 3 were recorded at a similar rate in both groups, while higher intensities (4 and above) were rare. No statistically significant differences in pain intensity were found (*p* = 0.933). Pulsatile pain remained the most frequently reported pain description in both groups (47.2% operated and 38.5% previously non-operated). Previously non-operated patients more frequently reported shooting pain (38.5% versus 13.9% operated), while sharp, wandering, and dull pains were present in comparable proportions in both groups. Burning pain was exclusively noted in the operated group (5.6%). Differences in pain descriptions were not statistically significant (*p* = 0.602). By the fourth day post-craniotomy, the prevalence of unexpected onset of pain increased to 72.2% of operated and 53.8% of previously non-operated patients. This increase may be associated with factors such as decreased efficacy of initial postoperative analgesia, patient movement, and the onset of inflammatory responses as part of the healing process. The difference in rates between operated and previously non-operated patients highlights the influence of recent surgical intervention and possibly accumulated experiences of pain management practices. Pain during movement was recorded in 13.9% of operated and 30.8% of previously non-operated patients, while pain at rest was rare in both groups (8.3% operated and 7.7% previously non-operated). Other times of pain occurrence, such as daytime (2.8%) or nighttime (2.8% operated and 7.7% previously non-operated), were less frequent. No statistically significant differences in the timing of pain onset were found (*p* = 0.576) (Table 5).

**Figure 2:**
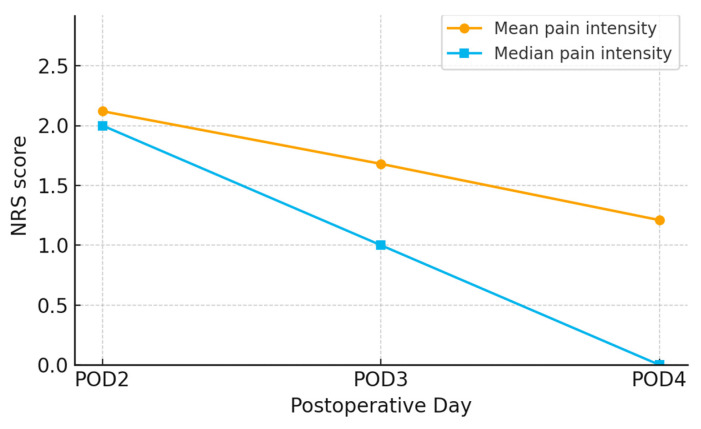
Pain intensity following craniotomy.

**Figure 3:**
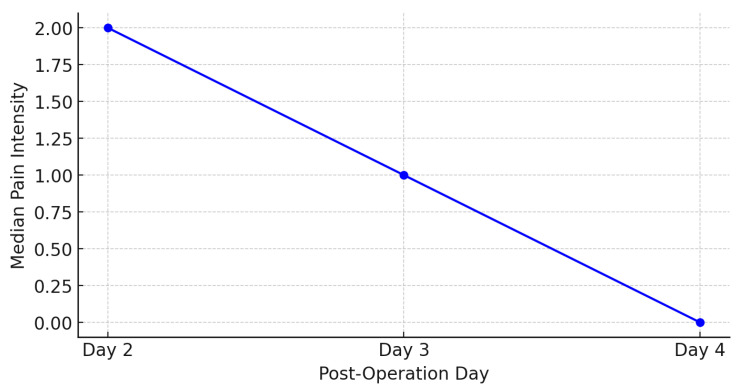
Trend of median pain intensity post-craniotomy.

The results from Figure 2 and Figure 3 show a trend of decreasing average pain intensity over time following craniotomy. On the second day post-operation, the average pain intensity was 2.12, which decreased to 1.68 on the third day, and further to 1.21 on the fourth day. The median pain intensity also shows a decline, with values of 2.00 on the second day, 1.00 on the third day, and 0.00 on the fourth day.

**Figure 4:**
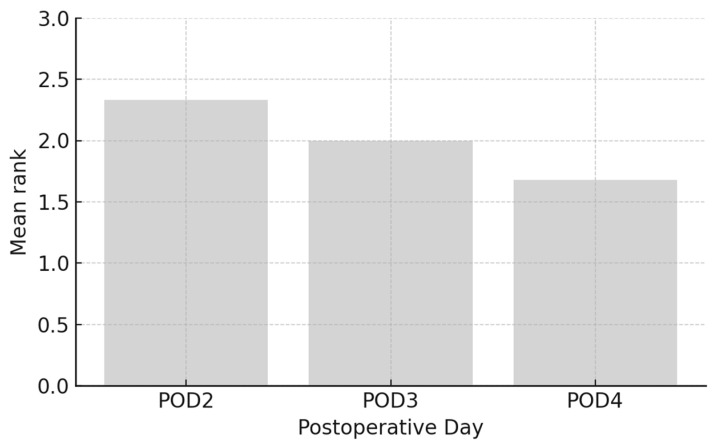
Pain intensity ranks following craniotomy.

The results from Figure 4 indicate a trend of decreasing arithmetic mean of pain intensity ranks over time following craniotomy. On the second day post-operation, the arithmetic mean of pain intensity ranks was 2.33, which decreased to 2.00 on the third day, and further to 1.68 on the fourth day. This trend suggests a gradual reduction in pain intensity as patients recover from the surgery.

The results of the Friedman test, shown in Table 6, indicate a statistically significant difference in pain intensity between the second, third, and fourth day post-craniotomy (*p* < 0.001). The Chi-Square value is 45.649 with 2 degrees of freedom, indicating that there is a significant change in pain intensity over time.

## 4. Discussion

The results of this study demonstrated a significant trend of decreasing pain intensity from the second to the fourth day following craniotomy. The average pain intensity decreased from 2.12 on the second day to 1.68 on the third day, and further to 1.21 on the fourth day. The median pain intensity also showed a reduction, from 2.00 on the second day to 1.00 on the third day and to 0.00 on the fourth day (Figure 2). The Friedman test confirmed a statistically significant difference in pain intensity across the days (*p* < 0.001) (Table 6). These findings suggest a natural recovery process and the effectiveness of applied pain management methods [8,9]. Recent studies have further emphasized the importance of structured postoperative care protocols in managing post-craniotomy pain. For instance, Abhinav et al. demonstrated that implementing an Enhanced Recovery After Surgery (ERAS) protocol significantly reduced pain intensity in patients undergoing elective craniotomies during the early postoperative period [16]. Additionally, Dean et al. provided a comprehensive overview of the various factors influencing post-craniotomy pain and highlighted the need for individualized pain management strategies [17]. A study conducted by Foust Winton et al. highlighted the reduction in pain intensity over the initial days following craniotomy, emphasizing the importance of continuous monitoring and adjustment of analgesic therapy [18]. Similarly, recent research has highlighted the efficacy of regional anaesthesia, such as scalp block, in reducing postoperative pain, aligning with our findings on the importance of adequate pain control [19].

Pulsatile pain was the most frequently reported characteristic in this study, consistent with literature describing this type of pain as predominant among patient’s post-craniotomy [6,8]. Various pain descriptions, including dull, sharp, and burning pain, indicate complex pain mechanisms, involving neuropathic components, especially in patients with larger surgical resections or manipulation of sensory nerves. It was observed that previously non-operated patients more frequently reported shooting pain, which could indicate different pain mechanisms depending on the type of surgical intervention or the presence of tumors (Table 4). Similar findings were recorded in a review article, where pulsatile pain was found to be the most common type of postoperative pain in patients after craniotomy, suggesting a vascular component of pain [20]. Moreover, the literature on Surgically Induced Neuropathic Pain highlights that various pain descriptions, including dull and sharp pain, may indicate the presence of neuropathic mechanisms, particularly in patients with larger surgical resections or manipulation of sensory nerves [21].

Pain triggered by activities such as movement, was strongest on the second day, with a gradual decrease in pain intensity by the fourth day (Table 5). These data confirm the recovery trend and underscore the importance of postoperative patient education on managing pain during physical activities. Similar findings were noted in a study by Kim et al., which found that patients post-craniotomy experienced the most intense pain during activities such as walking and coughing in the initial postoperative days, with a gradual reduction in pain by the fourth day [22].

Demographic data of the patients provided additional insights into this specific population. The most common tumors were gliomas (40%), meningiomas (32%), and metastatic tumors (18%), with vestibular schwannomas present in a smaller proportion (6%) (Table 2). The frontal lobe was the most common tumor location (28%), followed by the temporal (24%) and parietal lobes (16%) (Table 2). These results align with epidemiological data indicating the prevalence of these tumors in the adult population [23].

Contrary to expectations, no statistically significant differences in pain intensity were noted between operated and previously non-operated patients (*p* = 0.894) (Table 3). However, these differences in pain characteristics suggest individual variations in pain perception related to tumor anatomical locations and surgical approaches [8,24].

The most common tumor types in our sample were gliomas, meningiomas, and metastatic tumors, and the most frequent locations were the frontal and parietal lobes. Previous research has indicated that tumor location—particularly in the parietal and occipital regions—may influence postoperative pain due to greater edema or cortical involvement [25]. Future studies should explore the relationship between tumor characteristics and pain outcomes in more depth, ideally with imaging and surgical data. Recent studies suggest that a patient’s psychological state before surgery, particularly anxiety, can significantly impact the experience of pain after craniotomy. Patients with higher levels of preoperative anxiety often report intensified perceptions of pain postoperatively, underscoring the necessity for more tailored and aggressive pain management strategies to mitigate these effects [26]. One prospective observational study explored this relationship in patients undergoing scheduled craniotomy. Findings indicate that higher preoperative STAI scores are predictive of moderate to severe postoperative pain, emphasizing the importance of psychological preparation and targeted analgesia in managing post-craniotomy pain [10].

Compared to previous studies employing longitudinal designs or randomized analgesic interventions [16,18,19], our cross-sectional study offers a snapshot of pain trajectories during early recovery but does not capture long-term outcomes. While enhanced recovery protocols and regional blocks demonstrated strong analgesic effects in other studies [16,19], our unimodal regimen was more reflective of real-world, resource-limited settings. Furthermore, the use of a custom-developed questionnaire, while practical, may limit comparability with studies using validated pain scales.

Limitations of this study include a focus on short-term postoperative outcomes, which precludes analysis of the long-term effects of pain in this population. We also recognize the value of specifying surgical approaches to better understand their impact on postoperative pain; our study was limited in this regard as specific surgical details were not collected. Furthermore, although we collected data on tumor type and location, we did not analyze postoperative pain in relation to these variables, which limits conclusions regarding the influence of tumor characteristics on pain outcomes. Pain intensity was assessed solely with the NRS scale. While the NRS is simple and widely used in clinical practice, it is a one-dimensional and subjective tool that does not fully capture the complexity of pain, which should be considered when interpreting the results. Data analysis was performed by members of the research team who were aware of the study objectives, which may introduce potential bias due to the absence of blinded statistical analysis. Moreover, the study was not designed to compare the effects of individual analgesics, as drug administration followed routine clinical practice; therefore, no conclusions can be drawn regarding the impact of specific medications on postoperative pain perception.

Based on the findings of this study, future directions at our institution may include the development of a structured postoperative pain assessment protocol specifically for craniotomy patients, as well as the implementation of tailored pain management strategies. A follow-up longitudinal study is also planned to explore long-term pain outcomes and to evaluate the effectiveness of newly implemented protocols in a larger patient cohort.

## 5. Conclusions

In this study of patients undergoing craniotomy for brain tumors, postoperative pain was most intense on the second day and decreased rapidly by the fourth day. Pulsatile pain was the most frequently reported type. No significant differences were found between first-time and previously operated patients. These findings provide evidence that post-craniotomy pain in brain tumor patients follows a short-term declining trajectory, supporting the need for consistent monitoring and tailored pain management during the early postoperative period.

## Figures and Tables

**Table 1:** Sociodemographic data of patients following craniotomy for brain tumors.

		N	%
Gender	Male	51	51
	Female	49	49
	Prefer not to say	0	0
	Total	100	100
Age	Under 30 years	5	5
	30–60 years	62	62
	Over 60 years	33	33
	Total	100	100
Marital Status	Single	23	23
	Married	67	67
	Divorced	4	4
	Widowed	6	6
	Total	100	100
Occupation	Employed	55	55
	Unemployed	11	11
	Retired	34	34
	Student	0	0
	Total	100	100
Education	Postgraduate	5	5
	Higher education/University	36	36
	Secondary	54	54
	Primary	5	5
	Total	100	100

**Table 2:** Type and location of brain tumors and history of operations.

		Previous Surgery: Yes		Previous Surgery: No		Total		*p*
		N	%	N	%	N	%	
Type of brain tumor	Glioma	26	36.1	14	50	40	40	0.112
	Meningioma	26	36.1	6	21.4	32	32	
	Pituitary adenoma	0	0	1	3.6	1	1	
	Vestibular Schwannoma	2	2.8	4	14.3	6	6	
	Primary brain lymphoma	0	0	0	0	0	0	
	Medulloblastoma	1	1.4	0	0	1	1	
	Pineal region tumors	0	0	0	0	0	0	
	Craniopharyngioma	1	1.4	0	0	1	1	
	Metastatic brain tumors	15	20.8	3	10.7	18	18	
	Hemangioblastoma	1	1.4	0	0	1	1	
	Total	72	100	28	100	100	100	
Location of brain tumor	Cerebellum	7	9.7	1	3.6	8	8	0.462
	Frontal lobe	18	25.0	10	35.7	28	28	
	Frontal lobe, parietal lobe	2	2.8	2	7.1	4	4	
	Frontal lobe, parietal lobe, cerebellum	0	0	1	3.6	1	1	
	Frontal lobe, temporal lobe	5	6.9	0	0	5	5	
	Occipital lobe	6	8.3	1	3.6	7	7	
	Parietal lobe	11	15.3	5	17.9	16	16	
	Parietal lobe, occipital lobe	4	5.6	1	3.6	5	5	
	Parietal lobe, temporal lobe	1	1.4	1	3.6	2	2	
	Temporal lobe	18	25.0	6	21.4	24	24	
	Total	72	100	28	100	100	100	

**Table 3:** Comparison of pain intensity and characteristics on the second day post-craniotomy.

		Previous Surgery: Yes		Previous Surgery: No		Total		*p*
		N	%	N	%	N	%	
Pain intensity (second day after craniotomy)	No pain	29	40.3	12	42.9	41	41	0.894
	1	4	5.6	1	3.6	5	5	
	2	9	12.5	7	25.0	16	16	
	3	10	13.9	4	14.3	14	14	
	4	7	9.7	2	7.1	9	9	
	5	6	8.3	1	3.6	7	7	
	6	2	2.8	0	0.0	2	2	
	7	1	1.4	0	0.0	1	1	
	8	2	2.8	1	3.6	3	3	
	9	1	1.4	0	0.0	1	1	
	Unbearable pain	1	1.4	0	0	1	1	
	Total	72	100	28	100	100	100	
Pain description (second day after craniotomy)	Sharp pain	5	11.6	2	12.5	7	11.9	0.053
	Burning pain	5	11.6	0	0	5	8.5	
	Pulsatile pain	20	46.5	7	43.8	27	45.8	
	Colicky pain	0	0	0	0	0	0	
	Shooting pain	1	2.3	4	25	5	8.5	
	Wandering pain	5	11.6	0	0	5	8.5	
	Tightening pain	0	0	1	6.3	1	1.7	
	Dull pain	6	14	2	12.5	8	13.6	
	Pressure	1	2.3	0	0	1	1.7	
	Total	43	100	16	100	59	100	
Time of onset (second day after craniotomy)	Unexpected	24	55.8	5	31.3	29	49.2	0.240
	At rest	11	25.6	5	31.3	16	27.1	
	During movement	6	14	6	37.5	12	20.3	
	Shortly after eating	1	2.3	0	0	1	1.7	
	During the day	0	0	0	0	0	0	
	At night	1	2.3	0	0	1	1.7	
	Total	43	100	16	100	59	100	

**Table 4:** Comparison of pain intensity and characteristics on the third day following craniotomy.

		Previous Surgery: Yes		Previous Surgery: No		Total		*p*
		N	%	N	%	N	%	
Pain intensity (third day after craniotomy)	No pain	27	37.5	13	46.4	40	40	0.929
	1	12	16.7	5	17.9	17	17	
	2	10	13.9	4	14.3	14	14	
	3	12	16.7	4	14.3	16	16	
	4	3	4.2	1	3.6	4	4	
	5	4	5.6	0	0	4	4	
	6	1	1.4	0	0	1	1	
	7	1	1.4	0	0	1	1	
	8	1	1.4	1	3.6	2	2	
	Unbearable pain	1	1.4	0	0	1	1	
	Total	72	100	28	100	100	100	
Pain description (third day after craniotomy)	Sharp pain	4	8.9	1	6.7	5	8.3	0.350
	Burning pain	4	8.9	0	0	4	6.7	
	Pulsatile pain	20	44.4	6	40	26	43.3	
	Colicky pain	0	0	0	0	0	0	
	Shooting pain	4	8.9	5	33.3	9	15	
	Wandering pain	6	13.3	1	6.7	7	11.7	
	Tightening pain	6	13.3	2	13.3	8	13.3	
	Dull pain	1	2.2	0	0	1	1.7	
	Pressure	45	100	15	100	60	100	
Time of onset (third day after craniotomy)	Unexpected	30	66.7	5	33.3	35	58.3	0.067
	At rest	2	4.4	3	20	5	8.3	
	During movement	10	22.2	7	46.7	17	28.3	
	Shortly after eating	2	4.4	0	0	2	3.3	
	During the day	0	0	0	0	0	0	
	At night	1	2.2	0	0	1	1.7	
	Total	45	100	15	100	60	100	

**Table 5:** Comparison of pain intensity and characteristics on the fourth day following craniotomy.

		Previous Surgery: Yes		Previous Surgery: No		Total		*p*
		N	%	N	%	N	%	
Pain intensity (fourth day after craniotomy)	No pain	36	50	15	53.6	51	51	0.933
	1	14	19.4	6	21.4	20	20	
	2	9	12.5	4	14.3	13	13	
	3	5	6.9	2	7.1	7	7	
	4	4	5.6	1	3.6	5	5	
	8	3	4.2	0	0	3	3	
	Unbearable pain	1	1.4	0	0	1	1	
	Total	72	100	28	100	100	100	
Pain description (fourth day after craniotomy)	Sharp pain	5	13.9	1	7.7	6	12.2	0.602
	Burning pain	2	5.6	0	0	2	4.1	
	Pulsatile pain	17	47.2	5	38.5	22	44.9	
	Colicky pain	0	0	0	0	0	0	
	Shooting pain	5	13.9	5	38.5	10	20.4	
	Wandering pain	4	11.1	1	7.7	5	10.2	
	Tightening pain	2	5.6	1	7.7	3	6.1	
	Dull pain	1	2.8	0	0	1	2.0	
	Pressure	36	100	13	100	49	100	
Time of onset (fourth day after craniotomy)	Unexpected	26	72.2	7	53.8	33	67.3	0.576
	At rest	3	8.3	1	7.7	4	8.2	
	During movement	5	13.9	4	30.8	9	18.4	
	Shortly after eating	0	0	0	0	0	0	
	During the day	1	2.8	0	0	1	2	
	At night	1	2.8	1	7.7	2	4.1	
	Total	36	100	13	100	49	100	

**Table 6:** Test statistics for comparing pain intensity after craniotomy.

N	100
Chi-Square	45.649
df	2
Asymp. Sig.	0.000

## Data Availability

The data presented in this study are available on request from the corresponding author.
